# The Delivery of Health Promotion and Environmental Health Services; Public Health or Primary Care Settings?

**DOI:** 10.3390/healthcare6020042

**Published:** 2018-05-07

**Authors:** Lene Bjørn Jensen, Irena Lukic, Gabriel Gulis

**Affiliations:** 1 Public Health Consultant, Haderslev Municipality, Noerregade 41, 6100 Haderslev, Denmark; lbjj@haderslev.dk; 2 Unit for Health Promotion Research, University of Southern Denmark, 6700 Esbjerg, Denmark; irenalukic@hotmail.com

**Keywords:** public health operations, settings, decision tools, primary care

## Abstract

The WHO Regional Office for Europe developed a set of public health functions resulting in the ten Essential Public Health Operations (EPHO). Public health or primary care settings seem to be favorable to embrace all actions included into EPHOs. The presented paper aims to guide readers on how to assign individual health promotion and environmental health services to public health or primary care settings. Survey tools were developed based on EPHO 2, 3 and 4; there were six key informant surveys out of 18 contacted completed via e-mails by informants working in Denmark on health promotion and five face-to-face interviews were conducted in Australia (Melbourne and Victoria state) with experts from environmental health, public health and a physician. Based on interviews, we developed a set of indicators to support the assignment process. Population or individual focus, a system approach or one-to-one approach, dealing with hazards or dealing with effects, being proactive or reactive were identified as main element of the decision tool. Assignment of public health services to one of two settings proved to be possible in some cases, whereas in many there is no clear distinction between the two settings. National context might be the one which guides delivery of public health services.

## 1. Introduction

To identify the most important public health services and activities, several “essential public health functions” have been suggested over the years. In 1997, an international Delphi study produced a set of essential public health functions [1], which were modified by the Pan American Health Organization and the WHO Regional Office for the Western Pacific [2]. Adjustments to these essential public health functions have been developed by the WHO Regional Office for Europe (WHO EURO) and resulted in the ten Essential Public Health Operations (EPHO) [3], as follows:

1. Surveillance of population health and well-being.
2. Monitoring and response to health hazards and emergencies.
3. Health protection, including environmental, occupational, food safety, and others.
4. Health promotion, including action to address social determinants and health inequity.
5. Disease prevention, including early detection of illness.
6. Ensuring governance for health and well-being.
7. Ensuring a sufficient and competent public health workforce.
8. Ensuring sustainable organizational structures and financing.
9. Advocacy, communication and social mobilization for health.
10. Advancing public health research to inform policy and practice.

To each EPHO, a set of individual actions has been pre-defined by WHO EURO leading to question in which setting should those actions be conducted? Public health or primary care settings seem to be favorable to embrace all actions included into EPHOs, yet a recommendation on which action should be conducted where is not a simple task. 

The focus of public health lies in the health of populations and is concerned with all factors, which have an influence on the health of both groups of people and individuals [4]. Public health was defined by Acheson in 1988 as “the art and science of preventing disease, prolonging life and promoting health through the organized efforts of society” [5]. Natural disasters and the newly emerging infections have underlined the global responsibility of early coordinated responses [6] and with the beginning of the 21st century the importance of public health services and approaches has increased. The understanding of tasks and limits of public health services differ among European countries, as well as the extent to which public health is featured on national agendas. Despite the differences across countries, the focus in Europe has evolved in recent decades from sanitary provision and communicable disease control to the new public health which include health promotion, disease prevention and intersectional action. 

The concepts of primary care and primary health care have often been used instinctively in the literature, although they derive from different contexts. Historically, primary care is dated back to the United Kingdom (UK) in 1920, where it was intended for the regionalization of health services. Since then the concept has evolved and reached its potential with the establishment of UK National Health Services and the British model of general practice after World War II. These changes in the UK transcended into health systems throughout the industrialized countries and transformed into great variation [7]. The variety of notions used about primary care often refers to the level of health care services closest to communities or health care provided by health professionals at a person’s first point of entry into the health care system. For the public, at large in the industrialized world, primary care is mostly associated with medical care because the physician is their first point of entry to the health care system [7]. Primary care can be formally defined as “a multidimensional system structured by primary care governance, economic conditions, and a primary care development, facilitating access to a wide range of primary care services in a coordinated way, and on a continuous basis, by applying resources efficiently to provide high quality care, contributing to the distribution of health in the population” [8]. Primary care is profession centered and often only implies clinical contact [9].

Public health and primary care should be part of one health system [10]. The presented paper aims to guide readers on how to assign individual public health services to either public health or primary care settings. It covers health promotion and environmental health related services only, but generalizes findings in the discussion and conclusion part. 

## 2. Materials and Methods

A pilot study design was employed to develop the guidance to assign individual public health services to both or one of target settings. Denmark and Australia (Victoria state) were selected as study areas with interest to test the process in different health system settings. 

To select individual EPHOs and services enlisted under them we used the “Self-assessment tool for evaluation of essential public health operations in the WHO European Region (2015)” [11]. EPHO 4 “Health promotion” was selected direct as individual EPHO and services were edited with aim to shorten the survey tool. Environmental health services were gathered into one set from three EPHOs; EPHO 2, 3 and 4 and created a survey tool. The survey tools were discussed among authors and with an expert from clinical medicine who served as co-supervisor on health promotion services related analyses, but were not pretested. 

For health promotion services, there were 6 key informant surveys out of 18 contacted completed via e-mails by informants working in Denmark. On environmental health five face-to-face interviews were conducted in Australia (Melbourne and Victoria state) with experts from environmental health, public health and a physician. Respondents were asked to categorize individual services either to one of key potential provider settings or to both and justify their choice. 

## 3. Results

### 3.1. Health Promotion Services

The health promotion services that could be provided in agreement of respondents in primary care constituted of nine services. The major characteristic of these public health services is mostly related to services that are provided directly through patient contact. Most of the services require authorized health care professionals to be provided. These services are summarized in Table 1.

The health promotion services that could be provided in public health settings according to respondents are constituted by 22 services. The major characteristic of these public health services is mostly related to community work, inter-sectoral collaboration and information systems. These services are summarized in Table 2.

The public health services that could be provided in both a public health setting and primary care setting are constituted by 23 services according to respondents. This group contains both services where the public health setting does not apply, e.g., “management of moderate and severe acute malnutrition in infants and young children” and “intermittent supplementation of folic acid and iron for women in reproductive age”, and services where the primary care setting does not apply, e.g., “nutrition education, including food safety and physical activity, included in curriculum” and “Safe school environment for girls; skills-based education covering gender issues; promotion of girls’ education and empowerment”. Decision upon setting could be context based in different countries. Compared to agreement level on previous two categories (primary care or public health), the agreement level with these services was low. Summary of services is in Table 3.

### 3.2. Environmental Health Services

Table 4 summarizes categorization of service delivery places for environmental health services. 

In fact, only those broad services are assigned to public health where the five interviewees reached agreement. In those in “uncertain” they did not reached agreement, yet with exception of radiation control they categorized public health or either/or as delivery place. The services on radiation control are the only one where primary care has been mentioned by one interviewee direct. 

### 3.3. Indicators

Even more interesting as direct categorization of delivery places for individual health promotion or environmental health services are the indicators employed by respondents and interviewees to assign a delivery place. Summary of those decision tools is in Table 5.

## 4. Discussion

Looking at formal definitions of primary care and public health, there is a clear distinction. However, the distinction become less clear when looking deeper into what literature is explaining especially in terms of areas of responsibility and into practical routine work. Brown, Upshur and Sullivan [10] put the question “Public Health and Primary Care: Competition or Collaboration?” direct as title of their editorial. They conclude that public health and primary care should be two integrated parts of the same health system. The integration can for example be seen in the explanation of strong primary care, presenting preventive programs as part of strong primary care [12]. Literature also creates confusion in the differentiation of the terms primary care and primary health care. Primary health care includes both public health and primary care in its framework, placing primary care as the main setting, building the health care system around the primary care to create the health system that meets the need of all [13]. 

Even though the Health 2020 report in many ways is focused on strengthening public health, it recognizes primary health care as the center of service delivery [14]. In one instance, primary health care puts public health as the main actor, which is when focusing on public policies [13]. 

A major limitation of our study is the sample size and selection of two remote countries as study settings. The sample size is too small in both surveys to be able to make conclusions based on the results. One of the key barriers to get a larger sample size was the length of survey tool due to substantial listing of services. This also means that the results cannot be generalized, though it is a general question whether any results on the issue of public health—primary care can be generalized due to the significant role of national contexts. The results also must be seen in the light of the possibility of professional bias. To control for it, we tried to provide precise definitions of the public health setting and the primary care setting, but it seems like the respondents have not made sufficient use of the definitions. Respondents were likely to respond and categorize services according to their own professional background. In future research, noting the professional background of respondents and aiming to increase a balanced and most importantly larger sample size can lead to improved validity of results. 

However, the most important product of this research is the listing of indicators used by respondents to decide whether a service delivery is closer to a public health or primary care setting. Both the Danish survey respondents and the Australian interviewees used the same categories proving global generalizability of the presented categories. They can be of help also to national health policy makers while designing strategies and legislation on public health.

Despite all limitations of our pilot study, we believe that our findings place the EPHOs in slightly other perspective highlighting more the collaboration between public health and primary care. The EPHOs in addition to their original purpose to define essential public health services can be used also as an integration framework for settings providing the individual services in a specific governance and administration context. 

## 5. Conclusions

The presented research aimed to categorize health promotion and environmental health services of public health according to delivery setting, which was set as both or either a public health or a primary care setting. This proved to be possible in some cases, whereas in many there is no clear distinction between the two settings. Obviously, public health and primary care are both part of a health system and one of the key messages is, therefore, a need to coordinate the work of two settings rather then look for differences. National context is likely the one that guides delivery of public health services in a close collaboration with primary care. 

An important product of the research is a set of indicators, which can serve as decision tools while designing policies to provide all public health services. More research is needed on these indicators under specific national health system settings. 

In addition, and integrative perspective of EPHOs was identified as well. Despite being oriented on public health, a detailed analysis of services with focus on providing settings can identify different institutions inside and beyond a traditional health system. Future research should therefore include settings outside heath sector like for example municipalities, environmental directorates and other settings. 

## Figures and Tables

**Table 1:** Health promotion services provided in a primary care setting [11].

Provision of early childhood care, including regular check-ups, preventive services and healthy child development services
Screening and treatment of sexually transmitted infections
Access to fertility treatments
Access to safe medical and surgical abortion
Breastfeeding counselling and support in special-needs situations
Nutritional care and support for children living with HIV
Nutrition for children in an emergency context
Iron supplementation
Folic acid supplementation

**Table 2:** Health promotion services provided in a public health setting [11].

Empowerment of communities through local capacity-building, education, training and community mobilization
Community-based initiatives and partnerships
Establishment of information system, defining responsibilities and methodologies for data collection, analysis and use
Coherence of nutrition strategy with other policies related to health, agriculture, food safety, food industry, etc., information systems, monitoring and evaluation
Health promotion programs in community settings, including schools and workplaces
“Active transport” and urban development policies to promote walking and cycling, at the local and national levels
Efforts at a municipal or national level to ensure access to green space in urban environments
Communication campaigns to reduce obesity, including elements of diet and physical activity
Community-based strategies in sexual health education, including for vulnerable populations
Culturally sensitive communication campaigns to positively change social norms (on HIV, homosexuality, etc.)
Engagement with cultural and religious leaders to positively influence attitudes on sexual health
Quality of childbirth facilities, services and professionals
Information campaigns for the prevention of substance abuse, information systems, monitoring and evaluation
Performance of needs assessment research; generation of policy reports to obtain a comprehensive picture of mental health needs in the country
List of mental health services available within public health care system
Linkage with health and social services for prevention, detection, promotion and rehabilitation (including screening and prevention programmes for suicide and suicide risk)
Context-specific research on the causes of violence and effective prevention/protection strategies
Policies and programmes related to injury prevention, indicators and monitoring
Policies adapted to local conditions (urban versus rural, ethnic mix, gender issues, etc.) and developed in cooperation with local community leaders)
Strategy based on a critical analysis of the underlying causes for health inequities and identification of areas amenable to assessment
Development of information systems to track relevant target-based indicators, including income inequality, educational quality, access to healthy environments, employment opportunities, etc.
Measures aimed at building community support for health equity (e.g., through communication campaigns and awareness raising)

**Table 3:** Health promotion services to be provided both in primary care or public health settings [11].

Youth-friendly sexual health services
Ensuring broad access to information on the harm done by tobacco consumption, exposure to second-hand smoke and the benefits of quitting
Provision of direct support to smokers wishing to quit within the health care system, both in primary care and in specialized services
Increased capacity for prevention, treatment and care for all individuals and families affected by harmful use of alcohol
Specific programmes targeted to vulnerable groups
Dissuasive warnings on consumption of illicit alcohol to public
Facility- and community-level breastfeeding programmes/support
Maternity protection
Management of moderate and severe acute malnutrition in infants and young children
Intermittent supplementation of folic acid and iron for women in reproductive age
Nutritional support during emergencies for pregnant women
Nutrition education, including food safety and physical activity, included in curriculum
Specific food programmes for vulnerable populations (e.g., school lunch programme, food subsidies, etc.)
Programmes aimed at increasing intake of fruit and vegetables
Communication and educational programmes in community settings (health centres, workplaces, etc.)
Measures to identify and address malnutrition in adult and elderly populations
Family planning services
Linkage with health and social services for prevention, detection, promotion and rehabilitation (including screening and prevention programmes for suicide and suicide risk), monitoring and evaluation
Safe school environment for girls; skills-based education covering gender issues; promotion of girls’ education and empowerment
Use of reproductive/family planning services as entry points to support for victims
Research, analysis and dissemination
Defined roles in health and other sectors for a range of injuries and violence (poisoning, fires, drowning, falls, road traffic accidents, violence, etc.)
Public health approach followed (1) surveillance, (2) identification of risk factors, (3) development and evaluation, (4) implementation

**Table 4:** Division of environmental health services.

Primary Care	None
Public Health	Reducing air pollution Sanitation and drinking water Sanitation of swimming pools and public lakes Dust storms Bushfires, heatwaves and floods Indoor air quality Alert systems
Uncertain/either/or	Preparing for adaptation to impacts of climate change Land contamination Radiation inside and outside of hospital Reducing noise Indoor air pollutants Food safety both public and private spaces Investigation of disease clusters

**Table 5:** Decision tools.

Public Health	Primary Care
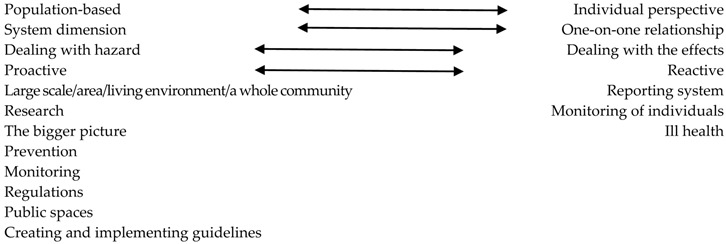	

The arrows signalize that the indicators were used with regard both settings, though in some case more often for primary care as for public health, or equally.
